# Beyond Statins: Emerging Evidence for HDL-Increasing Therapies and Diet in Treating Cardiovascular Disease

**DOI:** 10.1155/2018/6024747

**Published:** 2018-07-09

**Authors:** Sterling Farrer

**Affiliations:** Osteopathic Medical Student, Western University of Health Sciences, 22 Mullins Drive, Lebanon, OR 97355, USA

## Abstract

Coronary heart disease continues to be the leading cause of death in the United States. Current attempts to treat atherosclerosis and coronary artery disease often involve pharmaceutical and surgical treatments. While these treatments are successful in managing the pain from coronary heart disease, they do little to prevent or stop it. There are a number of clinical strategies that are currently being researched to treat atherosclerosis through HDL-increasing therapies. These clinical studies have shown positive effects through nutritional intervention, exercise, stress reduction, and tobacco and alcohol cessation. These treatment options are explored in greater detail, including their potential to halt and even reverse atherosclerosis. The results from these recent studies and how they relate to the mechanism of reverse cholesterol transport are also critically examined. Reverse cholesterol transport is a multistep process resulting in the net movement of cholesterol from peripheral tissues back to the liver via the plasma. The mechanism of reverse cholesterol transport is also further explored in this review.

## 1. Potential to Treat Atherosclerosis through Reverse Cholesterol Transport

The cost and health risks involved with cardiac stenting have turned physicians and researchers to explore alternative methods. Studies have shown success in treating atherosclerosis through preventive and lifestyle approaches. These approaches include nutritional intervention, exercise plans, and pharmaceutical intervention. In order to review and understand these methods and why they are successful, we must first understand how cholesterol is transported out of the arteries to stop the progression of atherosclerosis.

## 2. Mechanism of Reverse Cholesterol Transport

Our understanding of reverse cholesterol transport and its role in cholesterol efflux has progressed over the last few decades [1]. Reverse cholesterol transport (RCT) is a pathway by which cholesterol is transported from the artery walls to the liver for excretion from the body. It is through this process that the body reduces the amount of plaque buildup in vessel walls and reverses atherosclerosis.

HDL is synthesized in the liver and is a key factor in RCT. The liver releases the nascent HDL particles into the bloodstream. ATP-binding cassette transporter A1 (ABCA1) translocates cholesterol to the cell surface, where they appear to form lipid domains that interact with amphipathic *α*-helixes in apolipoproteins. ApoA1 and A2 on HDL stimulate the enzyme LCAT, which then esterifies the cell surface cholesterol molecules to form cholesteryl ester, which migrate to the core of the HDL particle to form mature migrating HDL[2]. Cholesterol esters are then exchanged for triglycerides in apoB100-containing lipoproteins (VLDL, IDL, and LDL which are further explained below). These cholesteryl esters are then taken up by the liver through the LDL-R Receptor. Finally, a liver protein named SR-B1 is the HDL receptor expressed on liver cells, allowing the liver to endocytose HDL, removing it from circulation [3].

LDL cholesterol also has an important function in the transport of cholesterol. Some cells cannot make enough cholesterol for the proper functioning of their membranes. LDL transports cholesterol made in the liver or sent to the liver from the intestine (as chylomicron remnants) to cells that need cholesterol. Thus, while the liver can express LDL-R to help regulate circulating LDL, almost any cell that needs cholesterol can express LDL-R.

Another lipoprotein particle released from the liver is very-low-density lipoprotein (VLDL). VLDL is released from the liver containing cholesteryl esters (CEs) and triglycerides (TGs). Once it enters the bloodstream, HDL transfers apolipoprotein C (apoC) and additional apoE to VLDL to become mature VLDL [4]. HDL also transfers cholesteryl esters to the VLDL in exchange for phospholipids and triglycerides via cholesteryl ester transfer protein (CETP). As more and more triglycerides are removed from the VLDL because of the action of LPL and CETP enzymes, the composition of the molecule changes, and it becomes intermediate-density lipoprotein (IDL) [4]. The composition of the IDL continues to change via CETP and LPL and it becomes LDL when the amount of cholesterol becomes greater than the amount of triglycerides. VLDL, IDL, and LDL all have the lipoprotein apoB-100 and can exchange triglycerides for cholesteryl esters from HDL [5]. These pathways show us that HDL interacts with several different lipoproteins, essentially taking the cholesterol from our vessels and transporting it to the liver for excretion.

## 3. High-Density Lipoprotein

Clinical studies have shown that congenital impairment in genes involved in cholesterol efflux may start atherosclerosis much earlier in life. The most commonly studied impairment that leads to higher plaque buildup is an imbalance in high-density lipoprotein (HDL) cholesterol levels. HDL has various species, identified on the basis of their major apolipoprotein (apo) components (apoA-I or apoA-II), density (HDL2 and HDL3), and electrophoretic mobility [6]. Studies have shown that low levels of HDL are associated with increased progression of atherosclerosis and risk of cardiovascular disease. Data from the Framingham Heart Study have shown that subjects with the highest HDL levels exhibit the lowest risk of developing heart disease [7]. Observational studies conducted throughout the world have consistently demonstrated that high serum levels of HDL are associated with reduced risk for CHD development and related complications such as myocardial infarction, stroke, and death, whereas low serum levels of this lipoprotein are correlated with increased risk for cardiovascular morbidity and mortality in both men and women [7]. The positive effects of HDL and its negative correlation with heart disease are thought to be mainly due to its primary role in reverse cholesterol transport.

Although the concept of reverse cholesterol transport from macrophages to liver and ultimately biliary excretion is the most popular mechanism to explain the ability of HDL to inhibit atherosclerosis, many other properties of HDL have been demonstrated in vitro that could contribute to its antiatherogenic effects [8]. In contrast to apoB-lipoprotein metabolism, the different components of HDL are largely assembled extracellularly and are subject to continuous dynamic exchange, transfer, and lipolysis within the plasma compartment [9]. These dynamic changes that HDL makes throughout the plasma help it to play an important factor in several processes, many which protect against atherosclerosis. In addition to reverse cholesterol transport, HDL may play a role in processes such as inhibition of endothelial inflammation, promotion of endothelial NO and prostacyclin production, and the sequestration and transport of amyloidogenic proteins, oxidized lipids, and lipids derived from exogenous pathogens [9].

## 4. Pharmacological Intervention

Considering that high-density lipoprotein cholesterol is a potent and independent epidemiologic risk factor and is a proven antiatherosclerotic agent, many attempts have been made to enhance HDL levels as a therapeutic approach for patients with atherosclerosis [10]. In prospective epidemiologic studies, every 1-mg/dL increase in HDL is associated with a 2% to 3% decrease in coronary artery disease risk, independent of low-density lipoprotein (LDL) cholesterol and triglyceride levels [11]. While our HDL levels are influenced by our genetics and lifestyle, pharmacological intervention has the potential to play an important role in enhancing our HDL levels. This option is much less well established, and the clinical endpoints are not as well investigated compared to the corresponding options for LDL-lowering drug therapies.

The most widely used drug to increase HDL levels is nicotinic acid or niacin. Niacin is thought to reduce the risk of cardiovascular disease by lowering LDL cholesterol concentrations and raising those of HDL cholesterol. Niacin is therefore often recommended to patients who have low HDL cholesterol concentrations. One study reports that niacin can increase HDL levels by 25% to 35%, when given the highest doses [12].

Despite niacin's apparent ability to increase HDL levels while also decreasing LDL levels, other studies have shown that niacin fails to reduce vascular events such as a stroke of any type or coronary or noncoronary revascularization [13]. In a large, randomized trial 25,673 participants were followed over a median duration of four years by the Clinical Trial Service Unit at Oxford University. The participants were men and women aged 50 to 80 years who had a history of myocardial infarction, cerebrovascular disease, peripheral arterial disease, or diabetes mellitus with evidence of symptomatic coronary disease. Participants took the combination niacin and laropiprant treatment for several weeks, and those who tolerated the drug combination were randomly assigned to receive two niacin/laropiprant combination tablets daily or matching placebo [13]. (Laropiprant is a drug used in combination with niacin to reduce the facial flushes induced by niacin. Laropiprant itself has no cholesterol-lowering effect.) As expected, the niacin tablets decreased patients LDL levels by an average of 10 mg/dL and raised HDL levels by an average of 6 mg/dL. However, assignment to treatment with niacin/laropiprant had no significant effect on the incidence of major coronary events and strokes. For both the placebo receiving group and the group receiving the niacin/laropiprant combination tablets, the rate of major vascular events was between 13 and 14%. The study did not specifically define major coronary events. The report did mention that coronary or noncoronary revascularization would be considered major vascular events; however, these are not necessarily the most relevant measures. Other specific endpoints such as MI and death would also be important to consider.

The niacin/laropiprant group, in comparison with the placebo group, was associated with a “highly significant excess of participants with fatal or nonfatal serious adverse events (7137 [55.6%] versus 6762 [52.7%]), with many participants having more than one serious adverse event,” the researchers reported [13]. The side effects included peptic ulceration, myopathy, skin related events, and even excesses of infection and bleeding, including gastrointestinal and intracranial bleeding.. While it is not noted in this study, it is a possibility that the discerning niacin effects may be due in part to laropiprant that was taken in combination with the niacin. The researchers wrote that the side effects could be due to the effects on glucose metabolism. Out of the participants receiving the niacin/laropiprant tablets, there was a 55% proportional increase in disturbances in diabetes control that were considered to be serious. In conclusion to this study, the researchers said, “Although niacin might still be relevant for particular patient groups (e.g., patients at high risk for vascular events who have high levels of LDL cholesterol), any potential benefits should be considered in the context of the observed hazards” [13]. Donald M Lloyd-Jones from Northwestern University Feinberg School of Medicine in Chicago also commented on this study by adding, “On the basis of the weight of available evidence showing net clinical harm, niacin must be considered to have an unacceptable toxicity profile for the majority of patients, and it should not be used routinely” [14].

Another drug that affects reverse cholesterol transport is ezetimibe. In a recent study, ezetimibe was shown to enhance macrophage reverse cholesterol transport in hamsters [15]. The hamsters showed a significant decrease in LDL cholesterol. Ezetimibe reduced tracer levels of ^3^H-cholesterol from prelabeled macrophages in the liver but increased them in feces, indicating promotion of reverse cholesterol transport in vivo. When a bile duct ligation was performed, it markedly inhibited macrophage-derived ^3^H-cholesterol excretion to feces and cancelled ezetimibe's stimulatory effect on RCT, suggesting that biliary cholesterol excretion is a major contributor in reverse cholesterol transport promotion by ezetimibe but the contribution of the transintestinal cholesterol efflux pathway is minimal. The researchers of this study concluded that ezetimibe exerts an additive antiatherogenic property by enhancing reverse cholesterol transport in hamsters and that this property is independent of the transintestinal cholesterol efflux pathway [15].

Similar to niacin, the evidence for prescribing ezetimibe as a primary lipid-lowering agent has not been shown to improve patient outcomes. The ENHANCE trial of ezetimibe and simvastatin was designed to show that ezetimibe could reduce the growth of fatty plaques in arteries [16]. It gave patients with genetically high cholesterol either statins alone or ezetimibe plus simvastatin. Then doctors measured the patients' LDL cholesterol levels and examined the patients' arteries to measure plaque growth. Adding ezetimibe to the statin did indeed reduce LDL cholesterol more than the statin alone did, but that did not improve the patients' arteries. In fact, after 2 years of therapy, the intima-media thickness had increased more in the ezetimibe/simvastatin group than in the simvastatin-only group, most notably in the most-diseased carotid and femoral segments, although the differences between groups were not statistically significant [16].

The study is not definitive and there may be several explanations of why the patients that took ezetimibe plus a statin had more plaque growth. These patients with high cholesterol because of genetics may not be representative of the entire population. Dr. Michael Davidson, a respected lipid expert but one invested in ezetimibe's development, says that the results of the ENHANCE trial can be explained away by the fact that most of the trial's participants had previously received lipid-lowering treatment, which obscured the effects of ezetimibe [17].

While these drugs have been successful in lowering LDL cholesterol and raising HDL cholesterol levels, the safety and efficacy of these drugs are yet to be determined. Definitive conclusions of the efficacy and safety of such drugs can be made at such a time when the results of more substantial and comprehensive trials are released. Further research is currently being done on these drugs, and further data on these trials will help doctors conclude whether these lipid-altering medications are an efficient strategy for treating atherosclerosis.

Another main concern with the pharmaceutical approach is the toll it is taking on America's healthcare costs. Atovorstatin, a commonly used statin or HMG-CoA reductase inhibitor, became the best-selling pharmaceutical in history in 2003. The manufacturer Pfizer reported sales of $12.4 billion in 2008 [18]. The high expense of these medications, as well as the increasing number of Americans suffering from heart disease, is a major contributor to the $312.6 billion that America spent on heart disease in 2011[19]. The American Heart Association predicts that this number will continue to increase, projecting that future costs will be around $444 billion a year [20]. These figures remain a substantial portion of our nation's healthcare expenditures.

## 5. Nutritional Intervention

One of the most intriguing areas of research within treating atherosclerosis and heart disease is nutritional intervention. Most doctors agree that diet is one of the most effective ways to prevent atherosclerosis. The American Heart Association have released specific diet guidelines to prevent cardiovascular disease. Their major guidelines are to consume a variety of fruits and vegetables and grain products, including whole grains, as well as including fat-free and low-fat dairy products, fish, legumes, poultry, and lean meats [21]. They also say to maintain a desirable blood cholesterol and lipoprotein profile by limiting the intake of foods with a high content of saturated fatty acids and cholesterol and to substitute grains and unsaturated fatty acids from vegetables, fish, legumes, and nuts [21].

Doctors and other researchers have taken this idea further, to treat and even attempt to reverse cardiovascular disease through aggressive nutritional intervention. One of the strongest advocates for this method is Dr. Caldwell Esselstyn from the Wellness Institute of the Cleveland Clinic. Dr. Esselstyn claims, “Though current medical and surgical treatments manage coronary artery disease, they do little to prevent or stop it. Nutritional intervention, as shown in our study and others, has halted and even reversed coronary artery disease” [22]. His study involved 198 volunteer patients with established cardiovascular disease. These patients transitioned from a usual diet to plant-based nutrition. They were only considered active participants if they completely refrained from dairy, fish, and meat. Whole grains, legumes, lentils, other vegetables, and fruit comprised the major portion of the diet.

Of the 198 participants, 177 were able to adhere to the plant-based diet. The researchers followed up with the participants over a 44-month period. In the group of 177 adherent patients, 112 reported angina at baseline and 104 (93%) experienced improvement of resolution of systems during the follow-up period. Major cardiac events judged to be recurrent disease totaled one stroke in the adherent cardiovascular participants, a recurrent event rate of .6% [22]. Thirteen of the 21 nonadherent participants experienced at least 1 adverse effect each, 2 sudden cardiac deaths, 1 heart transplant, 2 ischemic strokes, 4 PCIs with stent placement, 3 coronary artery bypass graftings, and 1 endarterectomy for peripheral arterial disease. There are a number of potential confounding variables to consider in these results. If one of the participants was hospitalized, they would not be able to choose their meals and would be placed into the nonadherent group. We must also take into consider that there is no control group in this study. All participants were volunteers, who had an interest in adhering to a plant-based diet. It would be beneficial to compare the results with a control group with established coronary artery disease that did not attempt to change their diet. Another confounding variable is that even though all participants had established coronary artery disease, the extent and severity of the disease varied for each individual. It is possible that the participants with the most severe coronary artery disease did not see any improvement of their symptoms and discontinued the diet. Further research with larger study groups and a randomized control group would be helpful to explain why the results of this study are so favorable.

Another study, published by the Atherosclerosis Journal, shows the positive correlation between vegetables and circulating endothelial progenitor cells [23]. In this study, forty-five healthy young women were employed and randomized to a dietary intervention group or a control group. Subjects in the intervention group received typical Okinawan vegetables through home-parcel delivery for 2 weeks. After the 2-week dietary intervention period, endothelial progenitor cells were significantly increased in the intervention group and were not in the control group. Changes in the endothelial progenitor cell number were inversely correlated with changes in both serum total cholesterol and low-density lipoprotein cholesterol level. The results suggest that green, leafy vegetables help to restore and rebuild healthy endothelium through increasing the number of endothelial progenitor cells. The results also establish a correlation between decreased numbers of endothelial progenitor cells and higher levels of serum cholesterol and LDL cholesterol levels, both risk factors of atherosclerosis and heart disease.

In another comparable study done in 1985, 22 patients with severe coronary artery disease were followed over a five-year period [24]. These patients took cholesterol-lowering drugs and followed a diet that derived no more than 10% of its calories from fat. The progression of their disease was angiographically documented by Dr. Ornish. These results were quantified with the percent diameter stenosis and minimal lumen diameter methods. Of the 22 participants, 5 dropped out within 2 years, and 17 maintained the diet, 11 of whom completed a mean of 5.5 years of follow-up. All 11 of these participants reduced their cholesterol level from a mean baseline of 246 mg/dL to below 150mg/dL [24]. Lesion analysis by percent stenosis showed that, of 25 lesions, 11 regressed and 14 remained stable. Mean arterial stenosis decreased from 53.4% to 46.2%. The mean decrease of arterial stenosis of 7.0% (P<.05) was greater than any reported in previous research. After the longitudinal study was complete, six patients continued this same diet and reported no further coronary events such as stroke or myocardial infarction. All five dropouts who resumed their prestudy diet reported new cardiac events. These included four instances of increased angina, two episodes of ventricular tachycardia, one coronary arterial bypass operation, one angioplasty, one case of congestive heart failure, and one death from complications of arrhythmia. In this study there was no specific control group noted. To more completely understand what specifically correlated with the lesion regression, it would be helpful to compare the lesion analysis to a control group that received the same cholesterol-lowering medication, with no change in diet. There are also a number of contributing factors that could have played a role in these results. The support system for this experimental group contributed to the patient's ability to stay on the diet. The social, psychological, or physiological factors from this support system may be more important for treating heart disease than the diet itself. A large, randomized control group would help to further correlate the diet with the results.

Other studies have analyzed more modest low-fat diets used in combination with drugs and achieved only partial success [25]. Dr. Esselstyn concludes, “ If the ultimate goal of treatment is total arrest of heart disease, it appears that the combination of less than 10% fat nutrition and cholesterol-lowering drugs is most likely to achieve the greatest reduction in serum lipids” [24].

Dr. Esselstyn suggests that it is the Western diet of added oils, dairy, meat, sugary foods, sugary drinks, refined carbohydrates, fruit juices, syrups, and molasses that set off the cascade leading to progressive endothelial injury, atherosclerotic plaque, and eventually cardiovascular disease. He adds that this “makes food choice a major, if not* the* major, cause of coronary artery disease. Future discoveries may help to explain why plant-based nutrition is so effective, yet we can postulate likely mechanisms. When foods that injure or cause endothelial dysfunction are avoided, the body readily restores the capacity of endothelial tissue to produce nitric oxide. Such change reduces production of vasoconstricting endothelin and thromboxane by injured endothelial cells” [24].

The question arises, is it reasonable to believe patients can maintain a diet of less than 10% fat nutrition throughout their lives? From the previous study we see that six patients continued to adhere to this strict diet, even though there were no longer being angiographically measured for the study. What were the factors for these six patients long term success?

Evidence from the Monel Chemical Senses Center, which studied three groups of volunteers who consumed different levels of dietary fat, suggests that people can lose their craving for fat. In the Monel study, only the patients whose diet contained less than 15% of calories from fat lost their desire for fat after 90 days [26]. This helps to make sense that more patients were able to continue to adhere to the 10% fat diet, rather than the more modest low-fat diets that were used in previous research. These findings also tell us that, in the first 90 days, the patient is in need of intense social support. There is early difficulty recognizing acceptable no-fat foods and dealing with the constant challenge of redesigning most traditional choices at every meal. In Dr. Ornish's experiment, a list of fat-free recipes taken from low-fat cookbooks and other resources on weight loss, cardiac health, and healthy lifestyle changes was given to each participant. For the initial several months, the constant challenge of shopping for appropriate foods and finding appropriate menus was a major focus [27].

There were also two other major factors that helped the participants adhere to the extreme change in their diet. Dr. Esselstyn credits Dr. Ornish by saying, “the physician had also adopted the diet and was thus a consistent role model for the participants. He actively involved himself in their care through frequent personal contact over a period of years and through periodic semisocial meetings that centered around the treatment plan. His personal investment in the success of his participants was clear to them. He was a credible source of information and was supportive of their efforts, especially through the more difficult initial stages of the study” [27]. The patients were also motivated by their initial weight loss, improved feeling of well-being, and decreasing angina.

In another study done at Tufts University, researchers concluded that healthier eating habits and a bit of discipline can help recondition the human brain to prefer healthy foods to junk foods [28]. The researchers found that people did not start out their lives with a love for French fries and other junk foods. People gain those cravings through eating it repeatedly. Susan B. Roberts, a professor of both nutritional science and psychology at the Tuft's Friedman School of Nutrition Science and Policy and Tufts University School of Medicine, studied MRI images of the brains of obese and overweight participants before and after completion of a six-month weight loss program. The results showed the areas of the brain associated with learning and addiction were transformed, with pleasure response centers becoming more sensitive to healthier food and less drawn to unhealthy, higher-calorie foods [28]. This supports the hypothesis that unhealthy habits are not necessarily fixed and that improved eating habits can be adopted and maintained. The findings suggest that we may begin to crave strict diets, such as the one used by Dr. Ornish to stop the progression of atherosclerotic plaque in his patients, if we condition our brain by repeatedly eating healthy foods.

Additional research supports the hypothesis that the Western diet of eating a high concentration of meat, oil, and dairy plays a large role in America's high rate of cardiovascular death. In other cultures and society where plant-based nutrition is prevalent there are extremely low rates of cardiovascular disease. In a paper authored by Strom and Jensen, they observed that in Norway between 1938 and 1948 there was a strong relationship between cardiovascular mortality and changes in intake of fat in the form of butter, milk, cheese, and eggs, with the changes in mortality lagging behind dietary changes by approximately one year [29]. From 1940 to 1945 Germany occupied Norway during World War II. During this time, German occupying forces confiscated their livestock, limiting Norwegians to plant-based nutrition. The death rate from strokes and heart attacks plummeted during this time. After World War II and the German occupation period, the Norwegians started eating animal based products again and the cardiovascular death rates increased as well.

The results from this study may correlate animal based products with cardiovascular disease, but there are a number of other explanations which could have contributed to these results. Another study observing the nutritional conditions during this time period in Norway points out other nutritional factors that have been shown to play a significant role in cardiovascular disease [30]. During the German occupation period, Norwegians calorie intake was 20% less than during the preoccupation period. Sugar intake was also cut in half during this time. Perhaps most significantly, fish consumption increased by 200%. There are a number of studies correlating fish intake with improved omega-3/omega-6 ratio and cardiovascular health.

Another well researched story that further supports plant-based nutrition is called “The Berry Project” and was designed in Finland to help farmers convert from dairy farming to berry farming. In 1973 Finnish men had the highest ischemic heart disease mortality rate in the world. The main focus of the strategy was to reduce the high saturated fat intake. Finnish villages were invited to participate in a cholesterol-lowering competition to demonstrate the feasibility of changing their diet and obtaining measurable, positive outcomes. The results were promising, as ischemic heart disease mortality decreased by 65% in the whole country from 1973 to 1995 [19].

Another positive effect that plant-based diets have on our health is its impact on our gut microbiome. Researchers have been continuing to unfold our understanding of the microbiome and its effect on human biology. As humans we have more bacteria than we do cell and gene count. The microflora has been found to have health promoting effects such as improved digestion, absorption, vitamin synthesis, and lowering of gas distension [31]. The good news is that we can alter our own microflora depending on the foods we eat. A study between two monozygotic Finnish twins found that diet played an important role in the modulation of their stool microbiota. The cotwins who ingested the same amounts of saturated fatty acids had very similar denaturing gradient gel electrophoresis profiles of* Bacteroides* spp., whereas the cotwins with similar consumption of fiber a very low bifidobacterial denaturing gradient gel electrophoresis profile similarity [31]. There are certain bacteria within the gut microflora that further breakdown the carnitine and choline found in animal products to trimethylamine (TMA). TMA is then transported to the liver where it is converted by an enzyme into trimethylamine N-oxide (TMAO). There are multiple studies published in 2013 that indicate that high levels of TMAO in the blood are associated with an increased risk of major adverse cardiovascular events [32]. A plant-based diet appears to select against gut flora that produce TMA by essentially starving the bacteria of carnitine and choline. One study had a group of vegans consent to participating in a study where they ate red meat and had their blood TMAO levels monitored. The study found that the formation of TMAO from the carnitine challenge was negligible in the vegans, compared to the control group, who had their TMAO levels drastically increase from eating the same amount of carnitine [33]. This concept explains the well-established link between high levels of meat consumption and cardiovascular disease risk. A recent article in the New England Journal of Medicine shows that choline in eggs, poultry, dairy, and fish produces the same toxic TMAO as carnitine in red meat [34]. This further explains plant-based protection from heart disease.

## 6. Effects of Exercise on Reverse Cholesterol Transport

While nutrition may be the most important lifestyle factor in stimulating reverse cholesterol transport, studies have shown that the intervention treatment is best when it is paired with exercise. One of the reasons for this is the positive effect exercise has on our HDL cholesterol levels. Epidemiological and clinical intervention data have consistently shown that low levels of HDL cholesterol are associated with an increased risk of cardiovascular disease. The first step in the management of low HDL cholesterol levels is to increase physical activity [35].

A study done in Czech Republic on obese women indicated that physical activity is required to trigger reverse cholesterol transport [36]. Efflux of cholesterol from prelabeled macrophages to plasma acceptors of tested individuals was used as a reverse cholesterol transport measure. Changes in reverse cholesterol transport were analyzed in 15 obese women after 9-week intervention, which consisted of 5 sessions of increased physical activity per week. Each session lasted 60 minutes long and 3 of the 5 were under controlled conditions in a fitness center. The other two sessions were usually bicycling or brisk walking. At the beginning of the 9-week intervention and at the end blood specimens were obtained.

At the end of the 9-week intervention the 15 obese women produced a substantial drop of body weight of more than 7 kg on average. The maximal body weight decrease was 15.5 kg whereas on the other side one volunteer decreased her body weight only by 2.3 kg. The significant finding of this experiment was the correlation between the amount of weight lost and the individual change of cholesterol efflux. Volunteers with the smallest weight reduction also displayed the smallest change of cholesterol efflux after intervention.

In another study including 155 sedentary men, the results showed that exercise can significantly change lipoprotein profiles [37]. In this study, 155 sedentary men were randomly assigned to one of the following three experimental conditions: weight loss by exercise, weight loss by diet, and control. To compare the experimental groups the researchers measured changes in the mass concentrations of subfractions within the LDL, IDL, and VLDL regions, changes in the size and buoyancy of the predominant LDL peak, and changes in the total mass concentrations of the HDL2 and HDL3 subfractions. These were measured at baseline, 7 months, and 1 year. There was increased mean HDL mass concentrations for both the diet induced and exercise induced weight loss groups. These results suggest that physical activity increases HDL levels as much, or possibly more, than dieting. HDL is one of the primary factors in inducing reverse cholesterol transport, making exercise another key component of preventing and treating cardiovascular disease.

Similar results were found in another study evaluating the main steps of reverse cholesterol transport in a group of well-trained soccer players in comparison to sedentary controls [38]. The capacity to promote cholesterol efflux from cells was significantly higher in the soccer players than in the control individuals (20.5% +/- 0.4% versus 15.9% +/- 1.2%, respectively). However, lecithin: cholesterol acyltransferase and cholesteryl ester transfer protein activities did not reach a statistically significant difference between both groups. Correlation analysis showed that cholesterol efflux induced by serum samples was directly related to HDL-C, HDL2-C, and lipoprotein A-I. On the other hand, negative correlations were observed with waist/hip ratio, low-density lipoprotein cholesterol, and apolipoprotein B. The researchers concluded, “the well-known cardioprotective benefit of regular exercise could be based, at least in part, on a less atherogenic lipid and lipoprotein profile and an enhanced cellular cholesterol efflux” [38].

Several studies have indicated that the most consistent effect of exercise on lipoprotein metabolism is an increase in high-density lipoprotein (HDL). A study published in the American Heart Association Journal investigated the effect of physical fitness on HDL and reverse cholesterol transport. The researchers studied several key steps in reverse cholesterol transport in endurance-trained athletes and compared them with a reference group of physically active individuals [39]. The 25 endurance-trained athletes were recruited from triathlon, biathlon, swimming, and running teams, while the reference group consisted of 33 normally active males. Plasma concentrations of HDL cholesterol and apoA-I were higher in athletes compared with controls. On average, HDL cholesterol was 21% higher and apoA-I was 13% higher in athletes. The concentration of LCAT was similar for athletes and control subjects; however, the activity of LCAT was 23% higher in athletes. Cholesterol efflux was tested as the capacity of plasma to promote cholesterol efflux from a macrophage cell line. Mean cholesterol efflux to plasma samples from athletes was 16% higher compared with that from controls.

In this same study, researchers also found that the trained athletes reached a threshold, where additional physical exercise did not correlate with higher HDL cholesterol levels. Dependence of HDL cholesterol and apoA-I on the level of aerobic fitness was linear up to a value of VO_2_max of 51 mL/min per kg; however, further increases in fitness were not accompanied by a further increase in HDL levels. This finding is consistent with conclusions of Durstine [40], who found a significant difference in HDL cholesterol between “recreational” and “good” runners, but not between “good” and “elite” runners. This may be linked to the threshold in the increase in muscle mass associated with increasing fitness.

Another important finding in this study was that higher levels of fitness were accompanied by higher concentrations of plasma pre*β*_1_-HDL. Pre*β*_1_-HDL is the first product of lipidation of minimally lipidated apoA-I and a likely acceptor of cellular cholesterol. Previous studies have suggested that pre*β*_1_-HDL may be a better marker of RCT than HDL cholesterol [41]. Pre*β*_1_-HDL is generated during passage of blood from artery to vein across human leg muscles and this can be substantially stimulated by exercise [42]. Because athletes have a bigger muscle volume, the researchers speculated that muscle may be an important source of higher pre*β*_1_-HDL levels in athletes [41].

## 7. Skepticism about the HDL Hypothesis

The cholesterol contained within HDL is known to be inversely related to cardiovascular disease; however, several failed clinical trials have created doubt HDL's ability to reverse atherosclerosis in patients. Several of these trials aimed at raising HDL cholesterol. Large outcome trials using niacin, fibrates, or cholesterol-ester-transfer-protein (CETP) inhibitors were performed but overall showed negative results or were only positive in subgroups [43]. These studies showed that simply raising HDL cholesterol will not reverse atherosclerosis. These negative studies were followed up with genetic studies, which indicated that low HDL cholesterol is not causally linked to atherosclerotic events [44]. A potential explanation for these inconsistent studies is due to the fact that HDL can be dysfunctional and lose their protective properties, such as in diabetes or inflammation. In periods of inflammation, apolipoprotein AI exhibits extensive posttranslational modifications through oxidative processes, particularly by myeloperoxidase, a peroxidase enzyme expressed in neutrophil granulocytes. The myeloperoxidase pathway inhibits cholesterol efflux causes HDL to lose its endothelial cell protective effects [45].

A potential focus of the future could be improving HDL functionality, rather than focusing on HDL concentration. As mentioned earlier in the text, studies involving exercise have not only increased HDL concentration, but also increased reverse cholesterol transport. Future studies could explore the different effects of exercise that could increase reverse cholesterol transport. For example, other studies have shown that increasing certain apolipoproteins, such as apolipoprotein AI, can induce plaque regression without a change in HDL concentration [46]. Discovering the exact mechanism of which weight loss and exercise improve HDL functionality will help explain why some HDL-increasing agents have not been shown to be clinically effective. Other medications that focus on the functionality of HDL may be more beneficial. For example, niacin has been shown to restore HDL functionality in diabetic patients and improve reverse cholesterol transport, without having a major impact on the concentration of HDL cholesterol [47]. However, the AIM-HIGH trial investigated simvastatin alone versus simvastatin with niacin on patients with established coronary artery disease and found that, among patients with atherosclerotic cardiovascular disease and LDL cholesterol levels of less than 70 mg per deciliter, there was no incremental clinical benefit from the addition of niacin to statin therapy during a 36-month follow-up period, despite significant improvements in HDL cholesterol and triglyceride levels [48]. The most likely explanation for these results is that, for such a population, already being aggressively treated with statins to lower LDL-C, it is hard to show an additional benefit. It could be possible that the additional niacin treatment would be more beneficial for a higher risk group with higher LDL concentration. Going forward, we have yet to establish a clinical translation for altering HDL concentration and function with medication. Several studies have shown the power of HDL to favorably modify plaque biology, but future studies need to further investigate the mechanistic base of HDL and focus on improving its function, rather than increasing its concentration. The potential of life style changes is often underestimated when it comes to improving the functionality of HDL. We have discussed studies that show diet and exercise treat atherosclerosis through reverse cholesterol transport, but as other clinical drug trials suggest, it is more complex than just improving the concentration of HDL.

## 8. Concluding Remarks

In this review of clinical strategies to use reverse cholesterol transport to stop and reverse atherosclerosis, I have evaluated the current medical approach to atherosclerosis and coronary heart disease in the United States. The present cardiovascular medicine approach can neither cure the disease nor end the epidemic and is financially expensive. The United States spends far more money on procedures and medication to fight against the cardiovascular disease epidemic than any other country, yet the number of Americans suffering from this disease is increasing each year. An aggressive lifestyle medicine approach treats cardiovascular disease through the body's natural cholesterol efflux system, namely, reverse cholesterol transport. Physicians need to work together to implement a strict and specific program to treat patients with cardiovascular disease. When patients start to see the positive results and popularity of such programs, there will be a greater desire to change their lifestyle and reverse cardiovascular risk factors.

There is need for additional research on exactly which diet and exercise program will work best to induce reverse cholesterol transport and treat cardiovascular disease. Additional research will further convince physicians and hospitals of the potential of these methods in treating the root of the cause, rather than just managing the patient's symptoms. Furthermore, the fact that dietary interventions to treat cardiovascular disease have, so far, been studied mostly in small clinical trials may increase physician's skepticism regarding the effectiveness of these interventions. Accordingly, it will be important for future clinical trials to be adequately funded to have high statistical power and to support appropriate controls. This may require significant NIH or even congressional intervention, however, since the standard model of drug companies funding trials for the drugs they will profit from does not apply. In the meantime, the data is strong and sound enough that patients should at least be informed with the option of nutritional intervention.

While nutritional intervention may have great potential in treating and reversing cardiovascular disease, the data presented also suggests that other lifestyle changes have a strong correlation with reverse cholesterol transport through affecting HDL cholesterol. Each of these lifestyle changes are significant factors in treating the current cardiovascular disease epidemic in the United States. The data has shown that with the proper support and coaching, people are willing to make these significant changes in their lives. There are certainly many challenges remaining, to identify both the most effective diet and exercise approaches and to identify effective means to get patients to adopt and maintain these approaches in their lives.
